# Penicillin binding protein 4: an emerging *Staphylococcus aureus* therapeutic target

**DOI:** 10.3389/fcimb.2026.1940058

**Published:** 2026-10-22

**Authors:** Vijay Singh Gondil, Monica A. Stefaniak, Christian Melander, Paul M. Dunman

**Affiliations:** 1 Department of Microbiology and Immunology, University of Rochester School of Medicine and Dentistry, Rochester, NY, United States; 2 Department of Chemistry and Biochemistry, University of Notre Dame, Notre Dame, IN, United States

**Keywords:** antibiotic resistance, antimicrobial, osteomyelitis, penicillin binding protein 4 (PBP4), peptidoglycan synthesis, *Staphylococcus aureus*, β-lactams

## Abstract

*Staphylococcus aureus* penicillin-binding protein 4 (PBP4) is a non-essential, low-molecular-weight transpeptidase that contributes to peptidoglycan maintenance under conventional laboratory growth conditions. Several recent studies have suggested that the protein’s cellular functions have far-reaching consequences, directly affecting *S. aureus* success as a human pathogen. Herein, we review recent work implicating PBP4 as a modulator of a noncanonical PBP2A-independent methicillin-resistant *S. aureus* (MRSA) phenotype that extends to the most recently developed fifth-generation cephalosporins. Furthermore, we review emerging studies indicating that PBP4 is a previously unappreciated virulence factor that governs an essential step in osteomyelitis pathogenesis, namely the invasion of osteocyte lacuna-canalicular networks (OLCNs). Given the protein’s involvement in these two disease-related processes, we discuss PBP4’s potential as a non-traditional antimicrobial therapeutic target.

## 1 Introduction

*Staphylococcus aureus* is a major human pathogen that is a frequent cause of both community- and hospital-associated bacterial infections, ranging in severity from superficial skin infections to life-threatening pneumonia, endocarditis, osteomyelitis, and septicemia (Kot et al., 2020; Chambers and Deleo, 2009; Tong et al., 2015). Healthcare concerns are further exacerbated by the emergence of *S. aureus* antibiotic resistance to virtually all front-line antibiotics, which in turn, is associated with high rates of morbidity and mortality, prolonged hospital stays, and increased treatment costs (Zhen et al., 2020; Anderson et al., 2009). Historically, the β-lactam class of antibiotics has been among the most popular and effective anti-staphylococcal agents, due to their potent bactericidal activity, bioavailability, and patient tolerance (Bush and Bradford, 2016). The β-lactams bind to and inactivate the transpeptidase activity of a set of enzymes that are generically referred to as the penicillin-binding proteins (PBPs), which catalyze the final step of bacterial peptidoglycan biosynthesis (Dörr et al., 2019). Peptidoglycan (PG) is comprised of glycan strands of alternating *N*-acetylglucosamine (NAG) and *N*-acetylmuramic (NAM) acid subunits that are covalently linked by β, 1→4 sugar bonds. Additionally, PG architecture/integrity is maintained by crosslinked NAM stem peptide side chains. While NAG and NAM are well-conserved peptidoglycan subunits across bacteria, the stem peptide and their linkage are variable among bacterial species (Navarre and Schneewind, 1999; Kim et al., 2015). In *S. aureus* and many other Gram-positive bacteria, stem peptides are comprised of L-Ala-D-Iso-Gln-L-Lys-D-Ala-D-Ala, which are predominantly crosslinked by the incorporation of a pentaglycine bridge between the fourth D-alanine residue of the donor stem peptide and the third residue (L-Lys) of the adjacent acceptor stem peptide (**Figure 1**). PBPs catalyze PG NAG-NAM transglycosylation and/or stem peptide crosslinking (Zapun et al., 2008).

**Figure 1:**
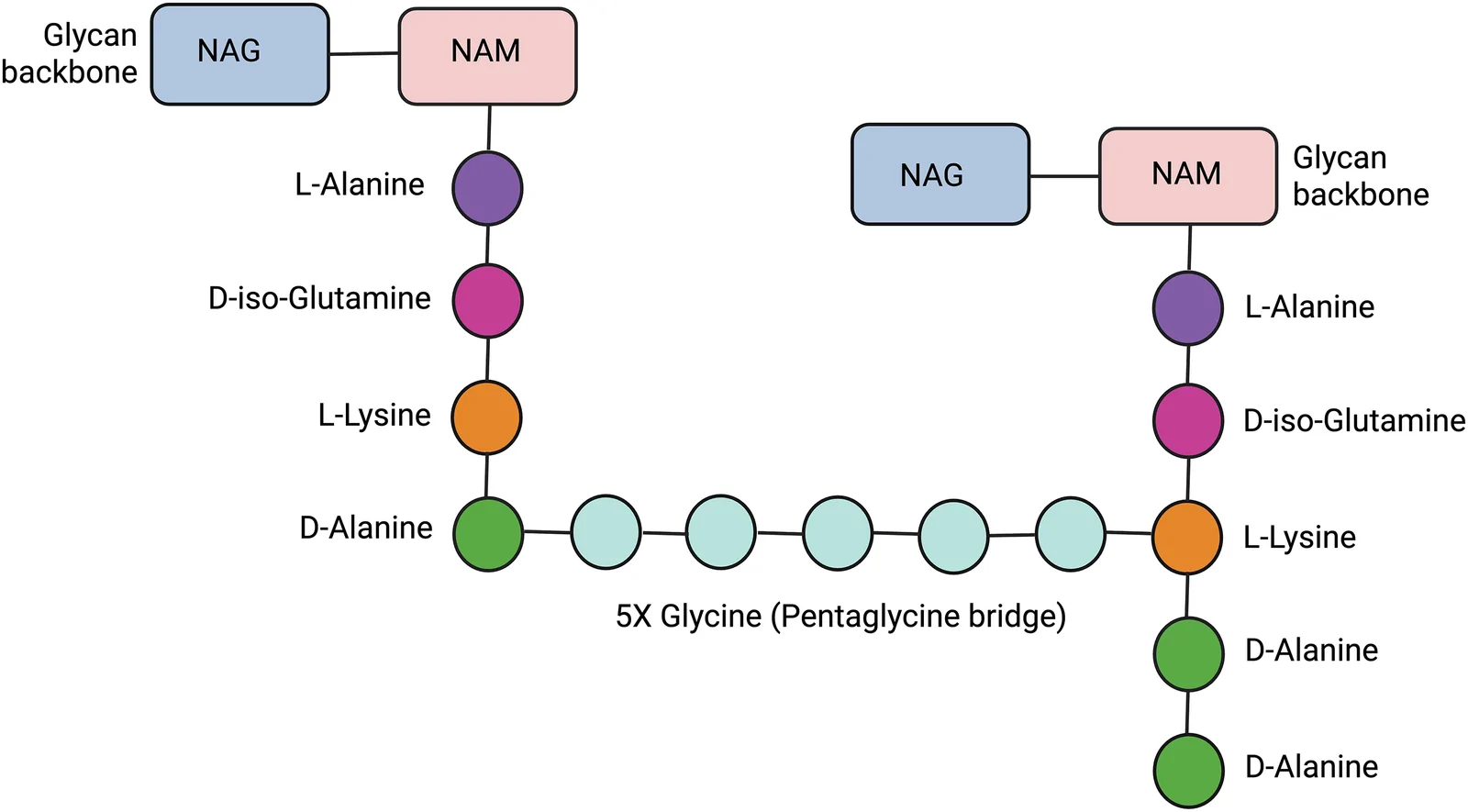
Schematic of *S. aureus* peptidoglycan architecture. Repeating disaccharide units of *N*-acetylglucosamine (NAG) and *N*-acetylmuramic (NAM) acid covalently linked by β, 1→4 sugar bonds. Stem peptide (L-Ala-D-Iso-Gln-L-Lys-D-Ala-D-Ala) is attached to NAM of the disaccharide unit and crosslinked *via* a pentaglycine bridge between the ϵ-nitrogen of L-lysine and the carbonyl carbon of D-Alanine of an adjacent stem peptide.

Bacterial PBPs can be divided into two main enzyme classes: high-molecular-weight (HMW; >45 kDa) and low-molecular-weight (LMW; <45 kDa) PBPs. HMW PBPs are multimodular enzymes comprising a cytoplasmic tail, a transmembrane domain, and an extracytoplasmic region and can be further subdivided based on their enzymatic activities and structural similarities (Giesbrecht et al., 1998). Class A HMW PBPs are bifunctional enzymes that confer both glycosyltransferase activity needed for NAG-NAM polymerization and transpeptidase activity needed for stem peptide crosslinking, whereas Class B HMW PBPs only exhibit transpeptidase activity (Murray et al., 1998; Spratt, 1977; Yanouri et al., 1993). Low-molecular-weight PBPs, also known as Class C PBPs, comprise a collection of enzymes that are largely dispensable for bacterial survival under conventional laboratory culture conditions but are thought to modulate cell shape and PG structure in response to stress. Hence, although they are non-essential for growth under laboratory culture conditions, LMW PBPs may be indispensable for bacterial survival in the host setting. LMW PBP activities vary, but members commonly include DD-carboxypeptidases that remove the stem peptide terminal D-alanine residue, peptidyl transferases that exhibit DD-transpeptidase activity, and endopeptidases that hydrolyze PG peptide cross-bridges (Popham et al., 1999; Severin et al., 1992; Sauvage et al., 2008).

The number of PBPs varies significantly among different bacterial species. For instance, the model Gram-positive organism, *Bacillus subtilis*, codes for 16 PBPs (4 Class A HMW, 6 Class B HMW, and 6 Class C LMW), whereas the model Gram-negative bacterium *Escherichia coli* codes for 12 PBPs (3 Class A HMW, 2 Class B HMW, and 7 Class C LMW). Such high numbers and functional redundancy can complicate studies aimed at understanding the biological contributions of individual PBPs in these organisms; nonetheless, in-depth experimental studies have advanced understanding of the cellular functions of many *B. subtilis* and *E. coli* PBPs. By contrast, *S. aureus* encodes 4 native PBPs (1 Class A HMW, 2 Class B HMWs, and 1 Class C LMW), making it a popular choice for studying penicillin-binding proteins and for understanding the mechanism(s) of action of antibiotics that disrupt cell wall synthesis. Nonetheless, despite this popularity, our understanding of the functional cellular roles of *S. aureus* PBPs is still emerging.

## 2 *Staphylococcus aureus* penicillin binding proteins

*S. aureus* PBP1 is an essential Class B HMW transpeptidase that is predominantly localized to the cell division septum (Pereira et al., 2007). The protein’s C-terminus confers transpeptidase activity and contains two PASTA (penicillin-binding protein and serine/threonine kinase-associated) domains that facilitate the protein’s interaction with cell division components and, consequently, cell division (Wacnik et al., 2022; Pereira et al., 2007; Reichmann et al., 2019). Recent studies have revealed that PBP1 pairs with a transglycosylase at the division septum, FtsW, which is likely to mediate NAG-NAM polymerization, while PBP1 facilitates stem peptide crosslinking activity needed to assemble daughter cell PG (Reichmann et al., 2019). Studies have established that PBP1’s role in recruiting and/or modulating FtsW activity, as opposed to the protein’s PG transpeptidase activity, accounts for its essentiality (Reichmann et al., 2019; Pereira et al., 2007; Taguchi et al., 2019). However, a more recent study revealed that either deletion of the PBP1 PASTA domain or mutation of the PBP1 transpeptidase catalytic Ser314 residue results in a loss of growth phenotype, suggesting that both activities are required for viability, at least in certain strains and/or growth conditions (Wacnik et al., 2022).

*S. aureus* PBP2 is an essential Class A HMW PBP and the only *S. aureus* penicillin binding protein that is known to confer both transpeptidase and transglycosylase activities (Pinho et al., 2013; Lund et al., 2018). PBP2 is thought to play a central role in septal peptidoglycan assembly, as it is selectively recruited to and accumulates at the division septum during cell division, where it can catalyze PG glycan assembly and pentapeptide cross-link formation (Pinho and Errington, 2005; Sinha Roy et al., 2001; Puls et al., 2023). However, the recent finding that *B. subtilis* FtsW and RodA “flippases” that flip NAG and NAM substrates to the extracellular space are NAG-NAM transglycosylases that interact with monofunctional PBP transpeptidases to modulate PG biosynthesis at the septum and periphery, respectively, suggest that orthologs may be responsible for *S. aureus* NAG-NAM polymerization and have rekindled studies aimed at understanding the essential functional role(s) of *S. aureus* PBP2 (Meeske et al., 2016; Emami et al., 2017). To that end, atomic force microscopy of *S. aureus* cell wall architecture indicates that the organism can produce two distinct peptidoglycan layers: a core of highly ordered concentric rings sandwiched between randomly oriented outer layers that make up the bulk of the septum thickness (Straume et al., 2021). These distinct architectures may indicate two biosynthetic processes, and it has been proposed that the combination of PBP1/FtsW may synthesize core PG, whereas *S. aureus* PBP2 may be responsible for synthesizing secondary PG layers (Tinajero-Trejo et al., 2025).

*S. aureus* PBP3 is a non-essential Class B HMW transpeptidase that has only recently been appreciated to play an important role in peripheral peptidoglycan synthesis (Reichmann et al., 2019). More directly, in high-resolution microscopy studies, it was observed that *S. aureus* undergoes PG elongation perpendicular to the cell division septum during the cell cycle (Monteiro et al., 2015). It was subsequently found that elongation is mediated by RodA/PBP3 pairs, which can confer NAG-NAM polymerization and stem peptide crossing linking activities, respectively (Reichmann et al., 2019). While *S. aureus* double mutants harboring a deletion of *rodA* and *pbpC* (PBP3) do not display an aberrant growth rate, they are enlarged and less elongated than wild-type cells, suggesting that RodA/PBP3 directs PG sidewall synthesis. A survey of 2522 *S. aureus* genomes deposited in GenBank (as of the time of writing) revealed that PBP3 is present within all genomes and shares 99.13% amino acid sequence identity across all isolates. Such a high degree of conservation suggests that elongation or an alternative PBP3 function(s) may be essential for survival in more physiologically relevant and/or alternative growth conditions.

*S. aureus* PBP4 is a non-essential Class C LMW transpeptidase that, like PBP3, is highly conserved with 99.07% amino acid sequence identity across GenBank entries. The protein’s transpeptidase activity is thought to confer an additional degree of *S. aureus* crosslinking within preassembled PG, contributing to the organism’s rigidity/cell wall integrity. Indeed, Δ*pbp4* mutant cells show a higher degree of elasticity as compared to the parent *S. aureus* NCTC 8325–4 strain (Wyke et al., 1981; Chen et al., 2012). The finding that PBP4 exhibits transpeptidase activity on partially crosslinked PG has led investigators to suggest that PBP4’s cellular role may be to repair peptidoglycan defects (Qiao et al., 2014). As elaborated further below, PBP4 has recently garnered considerable attention due to its role in mediated *S. aureus* disease processes and antibiotic resistance. We refer the reader to an excellent recent review on the practical contributions of PBP4 in mediating antibiotic resistance among methicillin-susceptible and resistant clinical isolates (Satishkumar and Chatterjee, 2026). The present review builds on the role of PBP4 in mediating antibiotic resistance and as a virulence factor, with particular emphasis on its role in recurrent osteomyelitis. We further discuss the promise and liabilities of targeting PBP4 for antimicrobial development.

## 3 β-lactam antibiotics

Given the importance of PBPs in modulating bacterial PG glycan polymerization and stem peptide cross-linking, combined with their ease of accessibility by small molecules, and that they are unique to prokaryotes, PBPs have been a preferred target for antibiotic development (Lovering et al., 2012b; Matteï et al., 2010; Nikolaidis et al., 2014). To that end, the β-lactam class of antibiotics targets bacterial PBPs and has been heralded as one of the most significant healthcare advances of the 20^th^ century. As the name implies, members of this antibiotic class share a common β-lactam ring structure containing an amide and carboxy group, which mimics the nascent PBP transpeptidase substrate D-Ala-D-Ala within *S. aureus* stem peptides (**Figure 2**) (Zapun et al., 2008). β-lactams bind to the active sites of PBPs and form covalent acyl-enzyme complexes between the PBP active site serine residue and β-lactam carbonyl ring that hydrolyzes very slowly, resulting in the inhibition of PBP peptidoglycan transpeptidase activity (Beadle et al., 2001). The β-lactams have historically been among the most widely prescribed antibiotic classes due to their high bactericidal activity, safety, and patient tolerance (Chiriac et al., 2017). Indeed, in 2003, β-lactams accounted for 65% of the global antibiotic market share; however, the emergence and widespread dissemination of β-lactam resistance, particularly among *S. aureus*, has become a significant healthcare concern for the management of infections in both hospital and community settings (De Oliveira et al., 2020; Elander, 2003).

**Figure 2:**
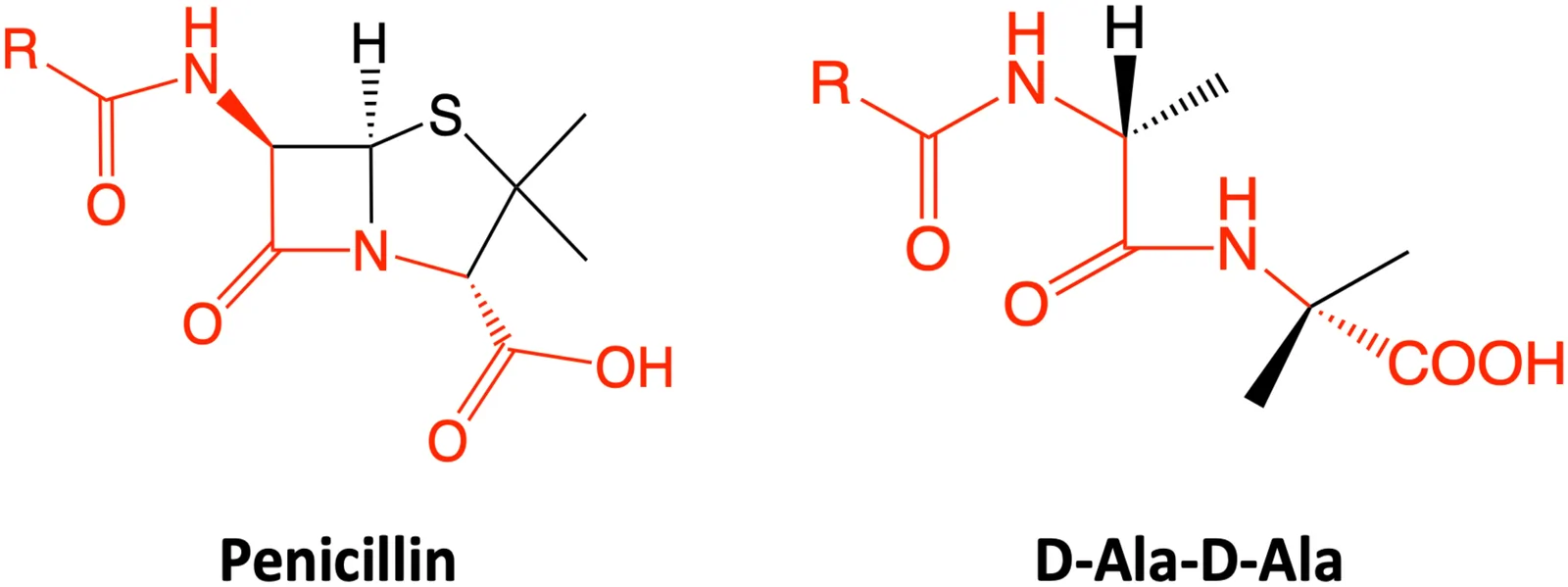
Structural similarity between penicillin and D-alanyl-D-alanine (D-Ala-D-Ala).

### 3.1 *S. aureus* β-lactam resistance

Penicillin was the first β-lactam antibiotic to be clinically used in 1943, thereby improving the prognosis for patients with staphylococcal infections. Yet, it was known as early as 1940 that bacteria could produce an enzyme that destroys penicillin, and a penicillin-resistant *S. aureus* strain was isolated from a hospital infection in 1942, one year prior to the introduction of penicillin for patient use (Abraham and Chain, 1940). Thus, it is not surprising that the healthcare benefits of treating *S. aureus* infections with penicillin were short-lived. As penicillin-resistant *S. aureus* emerged that harbored plasmid- or transposon- encoded *blaZ* penicillinase/β-lactamases that hydrolyze the penicillin β-lactam amide bond, the rapid dissemination of these hydrolases led to the demise of penicillin clinical efficacy (Lyon and Skurray, 1987). Indeed, by 1965, 50-82% of *S. aureus* clinical isolates were penicillin-resistant (Hawks, 1965). The emergence of β-lactamase producing *S. aureus* strains prompted the development of the semisynthetic β-lactamase-resistant/penicillinase-resistant penicillins, methicillin, oxacillin, dicloxacillin, and nafcillin (Bush and Bradford, 2016; Bamberger and Boyd, 2005). These newer generation penicillins retain the β-lactam core, but their bulky acyl side chains provide relative stability against hydrolysis by staphylococcal β-lactamases, thereby positioning them as a potential therapeutic option against β-lactamase producing *S. aureus* infections (Blanpain et al., 1980; Bush and Bradford, 2016). In addition, three penicillinase-resistant classes of β-lactam antibiotics were introduced to clinical practice: cephalosporins, monobactams, and carbapenems (**Figure 3**) (Pandey and Cascella, 2024). The cephalosporins are the largest and most frequently used class of β-lactam antibiotics. Members of this class contain a 7-aminocephalosporanic acid nucleus bonded to 3, 6-dihydro-2H-1, 3-thiazine rings and can be further subdivided into 5 generations based on their spectrum of antibacterial activity, with each iteration exhibiting improved efficacy against Gram-negative species and resistant strains (Chaudhry et al., 2019). Among these, the cephamycins are sometimes referred to as a new class of β-lactams but are often considered a new subclass of second-generation cephalosporins that contain a unique 7α-methoxy group, which confers broader β-lactamase resistance and a broader spectrum of antibacterial activity compared to other cephalosporins (Birnbaum et al., 1978). The monobactams are monocyclic β-lactam antibiotics that only affect Gram-negative PBPs, rendering them ineffective against Gram-positive pathogens (Finberg and Guharoy, 2012). Carbapenems are highly potent broad-spectrum β-lactam antibiotics characterized by a fused 4:5 ring structure in which the penicillin thiazolidine sulfur is replaced by carbon, contain a C2-C3 double bond, and a trans-hydroxyethyl side chain that contributes to β-lactamase stability (Papp-Wallace et al., 2011). The carbapenems are often considered a last resort for treating bacterial infections resistant to other antibiotics (Armstrong et al., 2021).

**Figure 3:**
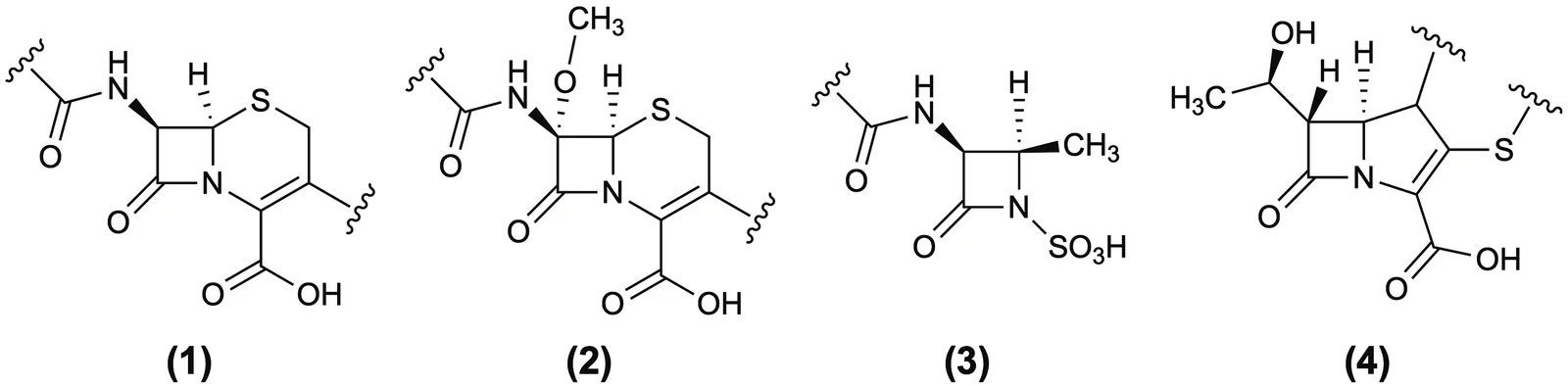
General structure of cephalosporins (1), cephamycins (2), monobactams (3), and carbapenems (4).

### 3.2 Methicillin-resistant *S. aureus*

MRSA was first described in 1961 and, as the term implies, refers to *S. aureus* strains that are resistant to methicillin, a β-lactamase-resistant, narrow-spectrum penicillin within the β-lactam class of antibiotics (Lee et al., 2018). While methicillin has been replaced by newer generations of β-lactam antibiotics and is no longer used clinically in the U.S., the term MRSA continues to be used generically to refer to broad β-lactam resistance (Dzintars and Grayson, 2018). Such resistance is rampant across the United States (Siddiqui and Koirala, 2024). Indeed, the Centers for Disease Control and Prevention reported that MRSA was responsible for 323, 700 nosocomial infections and 10, 600 deaths in the United States in 2017 (CDC, 2019).

Historically, the MRSA phenotype has been attributed to the acquisition of an auxiliary penicillin-binding protein, PBP2A, which is encoded by the *mecA* gene and carried on staphylococcal chromosomal cassette mec (SCCmec) elements. PBP2A is a Class B HMW transpeptidase penicillin-binding protein (Fishovitz et al., 2014). Like other *S. aureus* PBPs, PBP2A contains an active site serine residue at the N-terminal of the α2 helix that is critical for forming a PG peptide side chain acyl-enzyme complex needed for transpeptidase activity and is also the target site of β-lactams (Lim and Strynadka, 2002). However, in comparison to other *S. aureus* PBPs, the PBP2A active site is located within a narrow groove, which causes displacement and/or misalignment of β-lactam antibiotics (Lovering et al., 2012a; Fuda et al., 2004; Lim and Strynadka, 2002). In comparison to other *S. aureus* PBPs, PBP2A exhibits a slow β-lactam acylation rate constant (t_1/2_ 3–12 minutes) and a very high non-covalent pre-acylation dissociation constant (~600–1600 μM), presumably accounting for β-lactam recalcitrance (Fuda et al., 2004; Fishovitz et al., 2014). For resistance, it is believed that PBP2A functionally complements the transpeptidase activity of β-lactam-inactivated PBP2. More specifically, while β-lactams inactivate the transpeptidase activity of PBP2 (and other PBPs), PBP2’s transglycosylase activity is maintained. PBP2A complements the loss of PBP2 transpeptidase function, thereby allowing PBP2-mediated PG NAG-NAM polymerization and PBP2A-mediated stem peptide crosslinking in the presence of otherwise lethal β-lactam concentrations (Pinho et al., 2001). Thus, the presence of the *mecA* gene (PBP2A) has historically been synonymous with MRSA, and corresponding *mecA*-based detection methods have been employed for methicillin-resistant *S. aureus* surveillance studies. But as discussed below, such *mecA*-dependent diagnostics have probably underestimated the prevalence of MRSA.

Ceftobiprole and ceftaroline are the latest (fifth-generation) cephalosporins to be developed, and exhibit expanded PBP-binding and inhibitory spectrums; in addition to inactivating endogenous *S. aureus* PBPs, they also inhibit PBP2A and, thus, have been successfully used to treat MRSA. Ceftobiprole contains a vinylpyrrolidinone side chain, which facilitates the interaction of the molecule with the narrow groove of the PBP2A active site to position the lactam moiety within close proximity to the active site serine residue, thereby inactivating PBP2A (Lovering et al., 2012a). Ceftaroline is an allosteric PBP2A inhibitor that binds 60 Å from the protein active site, which in turn results in a conformational change that increases the active site volume (500-1300 Å), thereby allowing a second ceftaroline molecule to form a covalent acyl-PBP2A complex with the active site serine residue and inactivate the protein (Otero et al., 2013). Recently, intermediate to high-level ceftobiprole and/or ceftaroline-resistant MRSA strains have emerged, and studies of their underlying resistance mechanism(s) have suggested a previously unappreciated, PBP2A-independent MRSA pathway (Kelley et al., 2015; Nigo et al., 2017). More directly, among high-level ceftobiprole/ceftaroline-resistant strains, two mechanistic themes have emerged (Pinho and Errington, 2005; da Costa et al., 2018). First, fifth-generation cephalosporin resistance may arise by the accumulation of PBP2A mutations in both the transpeptidase and allosteric (non-penicillin-binding) domains, which diminishes ceftobiprole/ceftaroline binding and/or disrupts allosteric opening of the active site (Long et al., 2014; Otero et al., 2013; Kelley et al., 2015; Mendes et al., 2012). Second, as described in greater detail below, pioneering work by the Chambers and Chatterjee laboratories found that mutations leading to *S. aureus* PBP4 overexpression confer high-level resistance to ceftobiprole/ceftaroline and other β-lactams, thereby conferring a MRSA phenotype in a PBP2A-independent manner (Basuino et al., 2018; Masters et al., 2020; Hamilton et al., 2017).

## 4 *S. aureus* PBP4

As noted above, *S. aureus* PBP4 is a highly conserved, low molecular weight penicillin-binding protein that is non-essential for growth under conventional laboratory culture conditions. Most LMW PBPs are DD-carboxypeptidases that, similar to PBP transpeptidases, cleave the terminal D-Ala-D-Ala of the PG stem peptide via formation of an acyl-enzyme intermediate with the protein’s active site serine, resulting in D-Ala release. For carboxypeptidases, the acyl intermediate is subsequently resolved by H_2_O nucleophilic attack and results in the release of the tetrapeptide stem. In the case of transpeptidases, nucleophilic attack is mediated by the terminal amine of an adjacent stem peptide and results in PG crosslinking; such activity can be promiscuous as transpeptidases can also catalyze the exchange of non-canonical D-amino acids (NCDA) with the terminal stem peptide D-Ala, which is thought to regulate PG plasticity during stress conditions (Horcajo et al., 2012). Work primarily from the Tomasz, Chueng, and Walker laboratories indicates that *S. aureus* PBP4 is one of the few bacterial LMW PBPs that mediate cellular PG stem peptide crosslinking and NCDA exchange transpeptidase activities.

More directly, using high-performance liquid chromatography (HPLC), the Tomasz and Chueng laboratories were among the first to show that the cell wall composition of *S. aureus pbp4* (also known as *pbpD*) knockout strains displays a substantial decrease in PG stem peptide cross-linking in comparison to wild-type cells, indicating that PBP4 mediates cellular stem peptide transpeptidase cross-linking activity (Henze et al., 1996; Memmi et al., 2008; Łeski and Tomasz, 2005; Loskill et al., 2014) Moreover, Walker and colleagues subsequently showed that while wild-type cells readily incorporate fluorescently labeled D-amino acid substrates (NCDA) into their PG layer, such activity is radically reduced in *S. aureus pbp4* deletion strains, suggesting that PBP4 mediates PG stem peptide D-Ala transpeptidase exchange activity (Qiao et al., 2014). While such studies clearly indicate PBP4 impacts *S. aureus* cellular PG transpeptidase activity, they do not distinguish whether the protein’s effects are direct or indirect. To that end, HPLC studies of the PG composition of a *S. aureus pbp2* (also known as *pbpB*) conditional lethal mutant cell also showed a decrease of stem peptide crosslinking during PBP2 inhibitory conditions and that crosslinking was further decreased by *pbp4* mutation, leading the authors to conclude that PBP2 and PBP4 work in concert to catalyze PG crosslinking (Łeski and Tomasz, 2005). Thus, while it is probable that *S. aureus* PBP4 directly acts as a PG transpeptidase, it is also possible that PBP4 acts indirectly by recruiting other factors, such as PBP2, that catalyze PG peptide stem crosslinking and/or D-Ala exchange activity.

Perhaps the strongest evidence that *S. aureus* PBP4 directly confers cellular transpeptidase activity comes from *in vitro* biochemical studies by the Walker laboratory, combined with analyses of the protein’s amino acid sequence. Using purified *S. aureus* PBP4 lacking the transmembrane helix, it was found that PBP4 is capable of incorporating D-amino acids in muropeptide monomers and polymers despite the presence of excess concentrations of water, firmly establishing that, at least *in vitro*, *S. aureus* behaves as a PG transpeptidase, as opposed to a DD carboxypeptidase (Qiao et al., 2014). Moreover, analyses of the *S. aureus* PBP4 amino acid sequence suggest that the enzyme is a transpeptidase and allow predictions of the protein’s transpeptidase mechanism based on sequence conservation with better characterized staphylococcal penicillin binding proteins. The protein’s N-terminal amino acid residues (~25-310) encode a α-β-α sandwich and contains hallmark transpeptidase motifs common to other PBPs, which is followed by seven β-strands of unknown function (amino acids ~315–383) and C-terminal transmembrane domain (amino acid residues ~384–406) that are thought to be required for cell membrane anchoring (**Figure 4**) (Alexander et al., 2018; Navratna et al., 2010). The putative *S. aureus* PBP4 transpeptidase motifs include residues S75MTK (Ser-X-X-Lys; SXXK tetrad), S139SN (Ser-X-Asn; SXN triad), and K259TG (Lys-Thr(Ser)-Gly; KTG triad). Transpeptidase activity is thought to be catalyzed by nucleophilic attack of the Ser-75 γ-O by the carbonyl D-Ala-D-Ala amide bond of the PG peptide stem, allowing the formation of a tetrahedral intermediate before collapsing to form an acyl-enzyme intermediate and expelling the terminal D-Ala (Lovering et al., 2012b). Enzyme deacylation is thought to follow when the N-terminal moiety of the acceptor pentaglycine bridge acts as an acyl acceptor, resulting in peptide crosslinking between adjacent PG glycan chains (Lovering et al., 2012b). The SXN triad motif is likely to mediate proton transfer from the active site lysine to the leaving D-alanine amino group during acylation, thereby promoting peptide bond cleavage. The SXN triad is thought to act in concert with the neighboring KTG motif lysine residue, which activates the serine-lysine network, allowing SXN asparagine to deprotonate the incoming peptide amino group, thereby facilitating nucleophilic attack on the acyl-enzyme carbonyl during transpeptidation (Sauvage et al., 2008; Zapun et al., 2008). Admittedly, mechanistic studies designed to test these predictions remain in their infancy and, as noted above, are largely presumptive based on similar work involving other PBPs (Navratna et al., 2010; Qiao et al., 2014).

**Figure 4:**
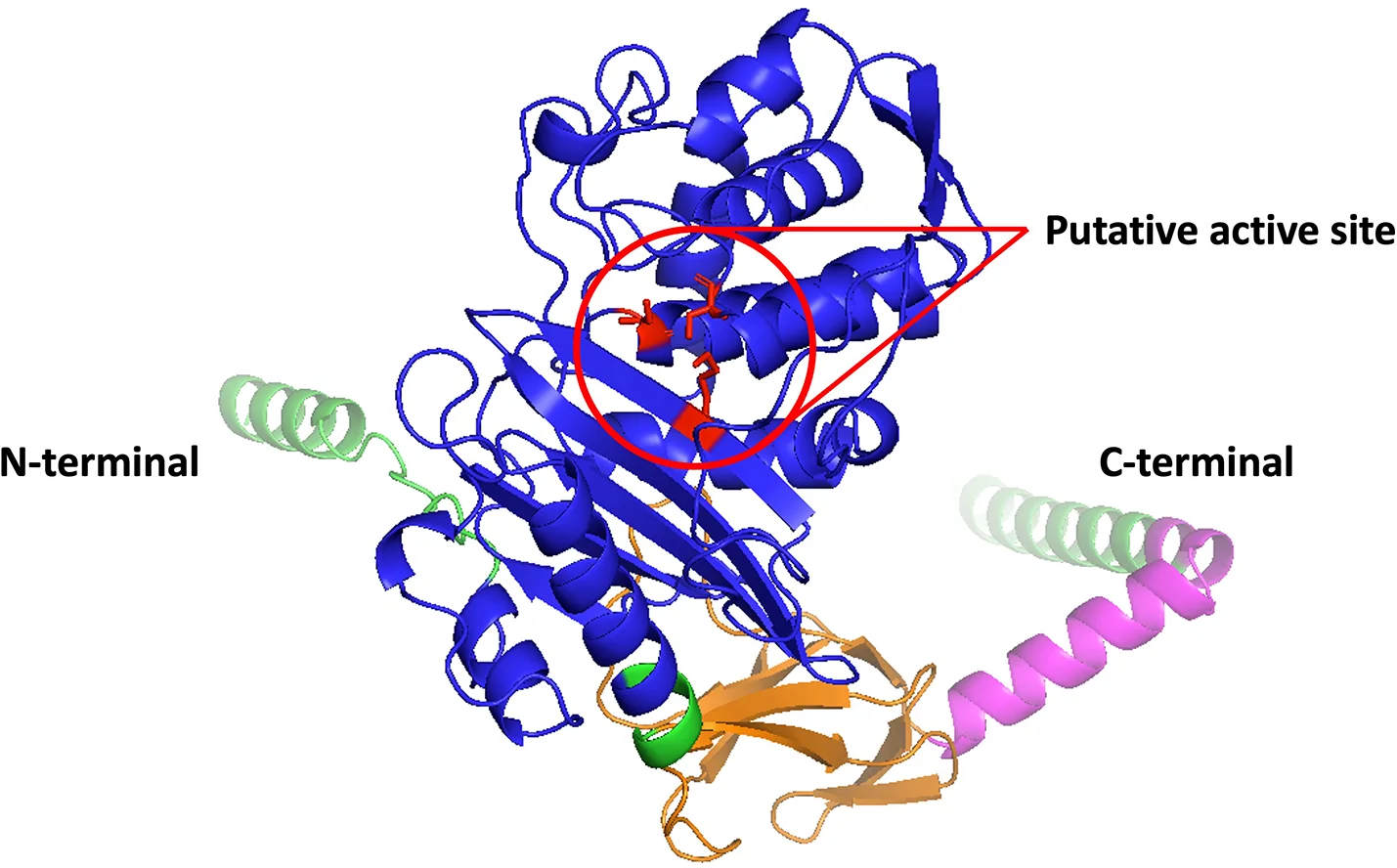
Structural characteristics of *S. aureus* PBP4. AlphaFold predicted structure of *S. aureus* PBP4. The putative N-terminal transpeptidase domain (α–β–α sandwich fold containing the active site) is shown in blue, the C-terminal β-barrel domain is shown in orange, and the transmembrane helix is shown in magenta. The presumed catalytic motifs S75MTK, S139SN, and K259TG are highlighted in red, clustered within the active-site cleft. The 3D structural model of *S. aureus* PBP4 was retrieved from the AlphaFold protein structure database (AlphaFold entry AF-A0A181GKX6-F1 and UniProt accession A0A181GKX6), colored, and annotated in PyMOL version 3.1.8.

### 4.1 PBP4-mediated β-lactam resistance

As noted above, historically, the presence of *mecA* (PBP2A) has been synonymous with an MRSA phenotype. Thus, it was surprising when several independent laboratories made the unexpected discovery that PBP4 mediates a non-canonical PBP2A-independent MRSA phenotype (Hamilton et al., 2017; Memmi et al., 2008; Basuino et al., 2018; Chatterjee et al., 2017). More directly, in a study designed to define the mechanism(s) of staphylococcal ceftobiprole/ceftaroline resistance, Chambers and colleagues performed stepwise-passaging of a *S. aureus mecA* knockout strain in the presence of increasing ceftobiprole concentrations and ultimately selected for a mutant that displayed high-level ceftobiprole resistance that was also highly resistant to other β-lactam antibiotics (ceftaroline and nafcillin) despite not being capable of producing PBP2A (Chatterjee et al., 2017). Whole-genome sequencing revealed that the resulting β-lactam-resistant strain contained a 36-bp duplication 290 bp upstream of the *pbp4* coding sequence and also contained two amino acid substitutions near the PBP4 predicted transpeptidase active site (Glu183-Ala and Phe241-Arg) (Chan et al., 2016; Banerjee et al., 2008; Hamilton et al., 2017). Moving the promoter mutation to the parent *mecA* knockout strain led to a 20–40 fold increase in PBP4 production and converted the otherwise β-lactam susceptible strain to ceftobiprole and ceftaroline resistant, suggesting that PBP4 overexpression is sufficient to confer a MRSA phenotype (Hamilton et al., 2017). To distinguish the effects of missense mutations on antibiotic resistance, steady-state studies of wild-type PBP4 and a PBP4 derivative harboring the E183A and F241R substitutions were performed, revealing that PBP4 containing the two substitutions exhibits differing β-lactam binding affinities depending on the antibiotic (Alexander et al., 2018). More directly, the missense mutations increased the protein’s ceftobiprole *K_m_* 150-fold but had no direct impact on ceftaroline binding (Alexander et al., 2018). Follow-up whole-cell studies have further evaluated the effects of the promoter mutation and two substitution mutations on PBP4-mediated resistance. While replacing the *pbp4* gene in a *S. aureus mecA* knockout strain with PBP4 E183A F241R did not confer β-lactam resistance, placing the promoter duplication upstream of the double mutant increased expression and enhanced β-lactam resistance (Chan et al., 2016; Basuino et al., 2018).

Similarly, Memmi and colleagues found that PBP4 governs β-lactam resistance within community-acquired MRSA isolates and were among the first investigators to propose that the protein may be a promising target for reversing antibiotic resistance (Memmi et al., 2008). More directly, in a study designed to investigate the contributions of non-essential *S. aureus* PBPs to MRSA, the team created strains harboring *pbp3* and/or *pbp4* deletions in various methicillin-resistant strain backgrounds (Memmi et al., 2008). Remarkably, while a loss of *pbp4* did not affect the resistance phenotype of hospital-acquired MRSA strains, *pbp4* deletion was associated with 16-fold reductions in β-lactam (oxacillin and nafcillin) resistance of community-acquired MRSA strains and could be complemented with wild-type *pbp4*. Furthermore, such reductions in antibiotic susceptibility were associated with reduced PG cell wall cross-linking, as expected of a PBP4-deficient strain background. To assess the therapeutic potential of putative PBP4 inhibitors on reversing MRSA phenotype, the team showed that treatment of 27 of 30 community-acquired clinical MRSA isolates with the PBP4-centric β-lactam, cefoxitin, completely reversed oxacillin resistance with strains unable to grow in the presence of <1 µg oxacillin (Memmi et al., 2008; Lade and Kim, 2023). It is currently unclear which genetic determinants modulate the observed differences in PBP4-mediated resistance between community- and hospital-associated MRSA isolates in that study, and these differences may be a byproduct of differing lineages or even SCCmec types; presumably, such studies are underway.

Clinical isolate surveillance studies have also linked PBP4 and the phosphodiesterase c-di-AMP regulator GdpP to an MRSA phenotype. More directly, genome sequencing of *S. aureus* clinical isolates has revealed that MRSA phenotypes do not explicitly depend on the presence of the PBP2A gene (*mecA*) (Göhring et al., 2011; Griffiths and O'Neill, 2012; Banerjee et al., 2010). Chatterjee and colleagues performed sequence analyses of 185 corresponding *mecA*-independent MRSA clinical isolates and found that 8 (4.32%) contained *pbp4* mutations, 22 (11.89%) contained *gdpP* mutations, whereas 155 isolates (83.78%) harbored mutations within the promoter region or open reading frame of both *pbp4* and *gdpP* (Lai et al., 2024). The overwhelming majority (101 of 163; 61.96%) of isolates with *pbp4*-associated mutants contained mutations within the gene’s promoter region and contained PBP4 amino acid substitution mutation(s), whereas 25 of 163 (15.33%) harbored mutations only within the *pbp4* promoter region, and 37 of 163 isolates (22.69%) contained only amino acid substitution mutations. Of the *gdpP* mutations, 128 of 177 isolates harbored stop codons or frameshift mutations, whereas 49% contained missense mutations. The authors went on to show that moving the *pbp4* promoter/*pbp4* substitution mutations into a clean *mecA*-methicillin-susceptible background increased resistance to the β-lactams nafcillin, oxacillin, and ceftaroline, whereas deleting the strain’s *gdpP* gene had minimal effect. Yet, the combination of the *pbp4* promoter/*pbp4* substitution mutation, together with knocking out the strain’s *gdpP* gene, synergistically led to high-level β-lactam resistance (Lai et al., 2024). While the molecular mechanism(s) underlying the synergistic resistance phenotype remain to be determined, these results indicate that *pbp4* overexpression, or that of a *pbp4* variant, plays a critical role in modulating the *mecA*-independent MRSA phenotype of *S. aureus* clinical isolates, irrespective of their *gdpP* genotype.

The association of PBP4 with β-lactam resistance is a relatively newly appreciated phenomenon. Hence, it is not surprising that the molecular mechanism(s) governing PBP4-mediated resistance have yet to be established. It is conceivable that the protein may act as a reservoir that binds and sequesters β-lactams, thereby lowering the active cellular antibiotic concentration. Alternatively, resistance could be attributable to the protein’s stem peptide- or D-Ala exchange- transpeptidase activity or to physiological perturbations associated with altered PBP4 activity, among other possibilities. Intriguingly, Navratna and colleagues solved the crystal structure of *S. aureus* PBP4 and suggested that the protein may act as a β-lactamase that inactivates β-lactams (Navratna et al., 2010). Indeed, the authors found that *S. aureus* PBP4 residues Gly181-Glu199 form a loop structurally similar to the Ω loop of- and are critical for- β-lactam hydrolysis by class A β-lactamases. Indeed, mass spectrometric analysis showed PBP4-mediated hydrolysis of ampicillin and cefotaxime, and the authors found that PBP4 elicits a structural change in the chromogenic cephalosporin nitrocefin; however, it remains to be seen whether this change is associated with nitrocefin forming an acyl-PBP4 complex, as is typical of most PBPs, rather than a released product, which would be expected of a β-lactamase (Navratna et al., 2010).

### 4.2 PBP4 and daptomycin tolerance

Growth in human serum is often used as a surrogate laboratory culture system to study bacterial physiology and virulence factors associated with sepsis. In one such recent study, Massey and colleagues found that PBP4 is associated with *S. aureus* serum-associated cell wall thickening and daptomycin tolerance in a panel of clinical isolates (Ledger and Massey, 2024). Further, serum-associated daptomycin tolerance could be reversed by the PBP4-centric β-lactam, cefoxitin, suggesting a direct role of PBP4 in modulating serum-induced *S. aureus* cell wall thickening and daptomycin tolerance (Ledger and Massey, 2024). To that end, increased cell-wall thickness and altered peptidoglycan architecture are associated with *S. aureus* intermediate vancomycin resistance (VISA) (Cui et al., 2021). Thus, it is intriguing to consider that this serum-associated PBP4-mediated daptomycin-resistant phenotype may extend to vancomycin and/or other antibiotic classes.

## 5 PBP4 plays a critical role in *S. aureus*-induced osteomyelitis

Osteomyelitis is an infection of the bone characterized by ensuing pathology, including inflammation, reduced blood supply, and bone destruction (Cram et al., 2012; Schwarz et al., 2019). Within the bone structure, bacteria are extremely difficult to eradicate, typically requiring two-stage surgical intervention requiring removal of the infected implant, debridement of the surrounding tissue, packing the infection site with antibiotic-laden beads, followed by implant replacement and long-term antibiotic treatment (Masters et al., 2019). Despite this aggressive approach, 20-30% of all osteomyelitis patients suffer recurrent infections and an amputation rate of 10-30% (Lipsky, 2004; Rosas et al., 2017). *S. aureus* is responsible for the majority (up to 60%) of osteomyelitis infections, and a long-standing question has been how the organism persists/cause recurring bone infections (Pimentel de Araujo et al., 2021; Tong et al., 2015; Zelmer et al., 2022). *In vitro* studies have suggested that *S. aureus* internalization by macrophages, osteoblasts, osteoclasts, and osteocytes may contribute to the pathogen’s long-term persistence in bone, but this has not yet been confirmed *in vivo* (Libraty et al., 2012; Masters et al., 2022). Recently, pioneering work by Schwarz and colleagues has identified an alternative source of *S. aureus* for recurrent infections, which is rapidly gaining acceptance as a predominant reservoir of reinfection.

Using scanning electron microscopy of chronically infected murine long bones and later diabetic patient bone, it was found that *S. aureus* readily colonizes the osteocyte lacuno-canalicular networks (OLCN) of the bone (de Mesy Bentley et al., 2017, 2018). OLCNs are a deeply interconnected network of 100–600 nm wide canaliculi in the mineralized bone matrix that are required for mineral homeostasis, mechano-sensing, and the mechanical properties of bone (Kollmannsberger et al., 2017). Interestingly, the average size of the OLCN is approximately 500 nm, whereas staphylococci are 1 μm; thus, the organism must deform to colonize the OLCN (**Figure 5**). A screen of a select transposon mutant library harboring disruptions of cell wall-associated genes revealed that PBP4 disruption leads to a loss of *S. aureus* OLCN invasion, as measured *in vitro* using Microfluidic-Silicon Membrane-Canalicular Arrays (µSiM-CA) that have been engineered and validated to model the OLCN orifice and within animals (Masters et al., 2020). Follow-up work revealed that within the OLCN, *S. aureus* is shielded from host defenses and antibiotics, thereby constituting a privileged reservoir for recurrent osteomyelitis and providing a direct route for invading canalicular network osteocytes and ensuing pathogenesis (Masters et al., 2021; Jensen et al., 2023). While the mechanism by which PBP4 contributes to OLCN invasion has not yet been established, atomic force microscopy elastic mapping studies have established that PBP4 contributes to the *S. aureus* elastic modulus and cell wall stiffness, which may be needed to overcome the sheer stress and/or maintain the cell wall integrity within the confines of the OLCN (Loskill et al., 2014).

**Figure 5:**
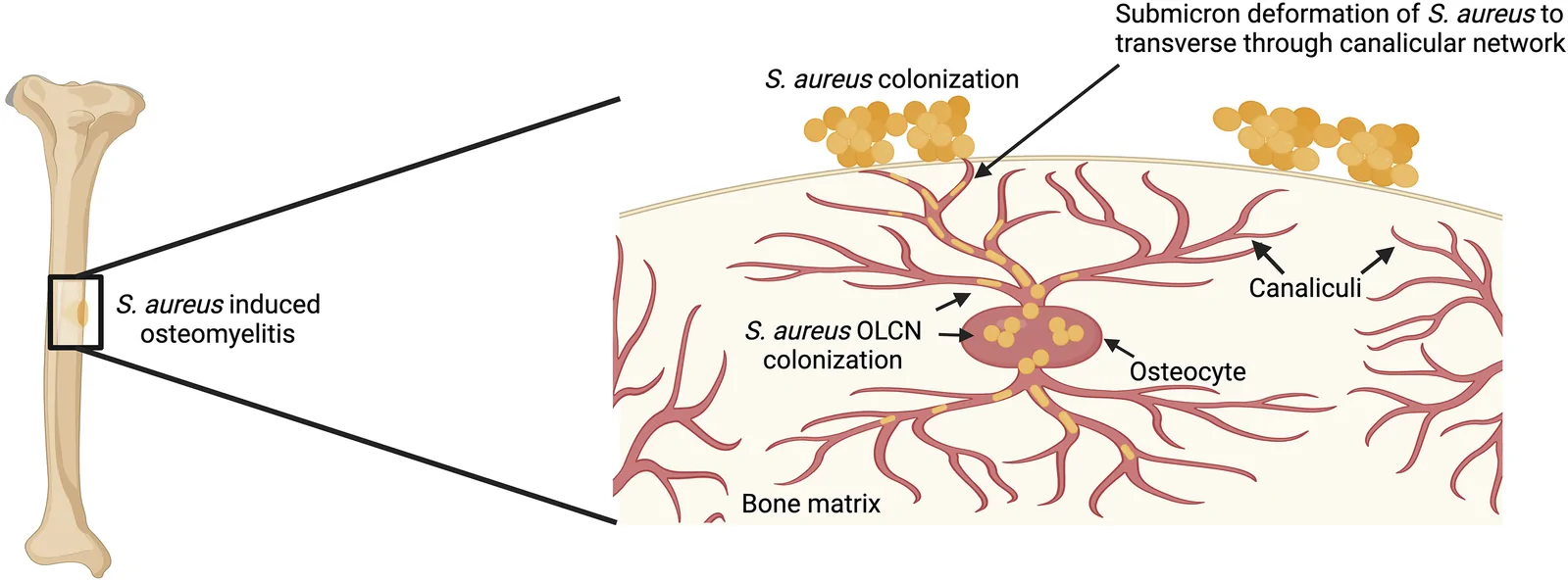
PBP4-mediated OLCN colonization may provide a privileged niche for *S. aureus* osteocyte invasion and recurrent and/or chronic osteomyelitis.

## 6 PBP4 as a target for antimicrobial drug development

Given the recent appreciation that PBP4 is associated with *S. aureus* β-lactam resistance, daptomycin tolerance, and OLCN invasion, several groups have suggested that it may be an attractive target for antimicrobial therapy. Such PBP4 inhibitors could ostensibly be used as antibiotic adjuvants that mitigate staphylococcal disease via two mechanisms. First, given that the fifth-generation cephalosporins, ceftobiprole and ceftaroline, are potent antimicrobial PBP inhibitors that inactivate PBP2A, but resistance can be achieved by PBP4 function, a combination of either antibiotic with a PBP4 inhibitor would be likely to break or reduce the emergence of *S. aureus* ceftobiprole/ceftaroline resistance and extend the lifetime of these two very important antibiotics. Second, given the critical role of PBP4 in mediating *S. aureus* OLCN invasion, a straightforward approach to preventing recurrent osteomyelitis would be to employ PBP4 inhibitors that could be co-administered during standard total joint replacement surgical prophylaxis and/or post-surgical antibiotic treatment regimens.

To that end, cefoxitin is a PBP4-centric β-lactam antibiotic that is widely used as a functional probe of PBP4-dependent processes at sub-antimicrobial concentrations due to its higher affinity for PBP4 in comparison to other *S. aureus* PBPs (Wyke et al., 1981; Okonog et al., 1995). Thus, cefoxitin may be an effective inhibitor of PBP4-associated antibiotic resistance and have clinical utility in combination with PBP2A active antibiotics, ceftobiprole, and/or ceftaroline. Indeed, it has been shown that PBP4-associated MRSA strains treated with subinhibitory concentrations of cefoxitin regain β-lactam susceptibility (Memmi et al., 2008; Ledger and Massey, 2024). However, cefoxitin does have liabilities that limit its effectiveness as a PBP4 inhibitor. Cefoxitin is not orally available and requires high-frequency intravenous dosing, which is associated with risks of nephrotoxicity, alterations in gut microbiota, and a high rate of β-lactam resistance among other bacterial species (Chabert et al., 2022; Mulligan et al., 1984). Further, because therapeutic doses of cefoxitin affect essential PBPs and, thus, are antimicrobial, it must be used prudently, as it can allow the outgrowth of bacterial species that are intrinsically resistant to the antibiotic and, simultaneously, select for resistance among otherwise susceptible species. For instance, empirical cefoxitin treatment has been associated with *Hemophilus influenzae* meningitis in pediatric populations (Feldman et al., 1980). Short-term cefoxitin use has also been shown to be associated with *Clostridioides difficile*-associated diarrhea (Block et al., 1985). Further, while the presence of a 7α-methoxy group makes cefoxitin a poor substrate for many β-lactamases, other β-lactamases, such as *Mycobacterium tuberculosis* Bla_Mab_, *Enterobacter cloacae* NmcA, and *Escherichia coli* CTX-M-9, can slowly hydrolyze cefoxitin, thereby conferring resistance (Soroka et al., 2013; Onishi et al., 1974; Chen et al., 2005; Matsuhashi and Tamaki, 1978). Thus, while cefoxitin is a PBP4 inhibitor, its antimicrobial effects will likely select for the acquisition of these resistance determinants and the outgrowth of intrinsically resistant bacterial species, which can be problematic. These limitations underscore the need for more selective, non-antimicrobial PBP4 inhibitor adjuvants.

Targeting non-essential proteins, such as PBP4, for antimicrobial therapy has certainly gained in popularity, owing in part to the presumption that there will be lower selective pressure for the emergence of resistance to inhibitors of non-essential targets compared to those of essential targets. However, this remains to be seen and, in fact, is probably the exception rather than the rule. Rather, it seems likely that a small molecule inhibitor of a non-essential protein that, for instance, plays a critical role in virulence or antibiotic resistance will select for outgrowth of mutants that merely exhibit reduced target binding, unless those mutations, themselves, inactivate the protein function. Likewise, there will likely be equal selective pressure and, hence, the outgrowth of mutants capable of reducing the inhibitor’s permeability, effluxing the inhibitor, chemically inactivating the inhibitor, and/or other target-specific bypass mechanisms. Thus, the resistance frequency of inhibitors of non-essential targets should be validated on a target-by-target basis, which, of course, could be addressed genetically but would be best investigated by identifying and characterizing such small-molecule inhibitors. A second plausible benefit of a selective, chemical inhibitor of a non-essential target is that its therapeutic use is likely to spare the normal flora. Indeed, it has long been known that disrupting the intestinal microflora with an antibiotic increases susceptibility to the corresponding drug-resistant bacterial infection (Bohnhoff et al., 1954). But here too, one must question the benefits realized, as it is difficult to imagine that perturbing one’s natural flora is an immediate/primary concern of a septic patient (or their healthcare provider). Even so, most bacterial vaccines target non-essential enzymes and volumes of studies that have validated their benefits, which, by extension, relegate “non-essential” targets as a valid antimicrobial development strategy, even if the benefits of doing so may sometimes be overstated.

To that end, *S. aureus* PBPs are recognized to be highly conserved cell wall enzymes with functional extracytoplasmic domains that modulate peptidoglycan synthesis and/or maintenance. As such they have been targeted for both active and passive vaccine development. Indeed, preclinical studies have shown that recombinant PBP2A vaccination elicits anti-PBP2A immune responses and protects mice against MRSA challenge, including reduced renal bacterial burden and improved survival (Haghighat et al., 2017). Similarly, prophylactic anti-PBP2A monoclonal antibody treatment has been shown to reduce *S. aureus* kidney burden and improve survival in a lethal murine model of infection (Saraiva et al., 2019). Thus, while *S. aureus* infection is often most problematic among immunocompromised patient populations it may be possible to target PBP4 using active and/or passive vaccine development. Small molecules represent another option for targeting *S. aureus* PBP4. In that regard, the active site of bacterial PBPs mimics the active site of β-lactamases and there are variety of well-known PBP inhibitors (i.e. the β-lactams) and β-lactamase inhibitors, and corresponding chemistry knowledge that may allow for focused medicinal-chemistry guided PBP4 selective inhibitor campaigns.

In a recent study, Young and colleagues empirically performed a high-throughput screen of a small molecule chemical diversity library to identify *S. aureus* PBP4 small molecule inhibitors that reverse the β-lactam resistance phenotype of cells that overexpress PBP4 E183A F241R to that of a *S. aureus Δpbp4* strain (Young et al., 2022). A total of 6 chemical classes were found to reduce and/or eliminate PBP4-associated β-lactam resistance. These agents did not impact bacterial viability in the laboratory setting, were confirmed to bind PBP4 over other PBPs selectively, and inhibited PBP4-associated incorporation of fluorescent D-Ala into *S. aureus* PG and OLCN invasion *in vitro*. The phenyl urea and benzothiophene classes of *S. aureus* PBP4 inhibitors have received the most attention thus far. Exploiting the *S. aureus* PBP4 crystal structure, in silico modeling was leveraged to engineer analogs with a higher likelihood of interfering with the putative PBP4 active site. Analogs of both chemical classes demonstrated greater ability to overcome PBP4-mediated β-lactam resistance, to inhibit D-Ala incorporation into *S. aureus* cell walls, and to limit OLCN invasion than the parental molecules (Gondil et al., 2024). Interestingly, the analogs of phenyl urea and benzothiophene PBP4 inhibitors were also found to reverse PBP2A-mediated β-lactam resistance, suggesting they may be more promiscuous PBP inhibitors that could target both conventional and non-canonical MRSA phenotypes (Butman et al., 2025; Stefaniak et al., 2026). The reason(s) for this change in PBP fidelity remain to be determined and highlight the need for continued study of *S. aureus* PBP4 and corresponding inhibitors.

## 7 PBP4 inhibitor clinical development considerations

Practically speaking, the goal of a clinical research program is to establish the safety of a therapeutic while simultaneously enrolling patients with a sufficiently high background event rate to confidently detect an efficacious effect, while also balancing a reasonable patient sample size, enrollment time, and cost. In terms of the clinical development of a PBP4 inhibitor (iPBP4) for the prevention and/or treatment of *S. aureus-*associated osteomyelitis, one could ostensibly assess the efficacy of an iPBP4 when combined with standard-of-care antibiotic prophylaxis treatment regimen(s) that are given to individuals undergoing elective total joint replacement (TJR). In this scenario, the antibiotic would be expected to reduce bacterial contamination, whereas the iPBP4 would act as an adjuvant to reduce OLCN invasion by *S. aureus* that has escaped antibiotic treatment and, consequently, further reduce corresponding TJR-associated osteomyelitis. While there are approximately 1.5–2 million TJR annually in the U.S., the incidence of corresponding TJR-associated osteomyelitis is relatively rare, occurring between 1% and 2% of patients, due, in part, to advances in aseptic techniques and surgical practices (AAOS-2025, 2025; Trudeau et al., 2026; Osmon et al., 2013). Thus, the number of resulting osteomyelitis cases annually is actually quite low (~10, 000 patients annually) and, in reality, it is probably not practical to develop a clinical development plan that is designed to measure the prophylactic effects of the standard of care +/- iPBP4 for TJR patients based on the sheer number of patients required in each treatment arm (antibiotic vs. antibiotic + iPBP4) to reasonably measure the ability of the iPBP4 to improve upon a ~1% infection rate. As an example, if one considers osteomyelitis infection rate of TJR patients to be 1% following standard of care antibiotic prophylaxis and subsequent treatment; addition of an adjuvant (iPBP4) that displays a 50% further reduction in osteomyelitis would be expected to require approximately 4, 673 patients per arm (antibiotic vs. antibiotic + iPBP4) or 21, 784 patients per arm (for a 25% reduction due to iPBP4) based on 80% power, two-sided test α = 0.05.

Another patient population that could be considered for iPBP4 efficacy measures includes the ~1% of TJR patients who do develop osteomyelitis. For this patient population, once finally effectively treated, the infection recurrence rate is approximately 16% (Piuzzi et al., 2025). Thus, such individuals effectively represent an enriched patient population that could be considered for clinical trials designed to measure the efficacy of therapeutics (or devices) intended to reduce the incidence of recurrent osteomyelitis, providing a reasonable opportunity to measure the efficacy of agents that reduce recurrence below 16%. Nonetheless, PBP4 mediated *S. aureus* OLCN colonization is thought to provide a reservoir for reinfection. Thus, for this group of patients, OLCN colonization has presumably already occurred, and it is unlikely that an iPBP4 would reduce infection recurrence rates unless the disease involves recurrent cycling of viable *S. aureus* between the OLCN and the bloodstream in a PBP4-dependent manner.

While TJR patient populations may not offer attractive clinical development paths for an iPBP4, a more practical patient population for evaluating iPBP4’s efficacy in mitigating osteomyelitis could be adults with severe open tibial fractures (Gustilo-Anderson Type IIIA or IIIB), as these individuals have a high incidence of osteomyelitis. Indeed, a retrospective study of 235 patients with Type III open diaphyseal tibial fractures undergoing standard-of-care antibiotic treatment found that 16.6% of patients developed osteomyelitis, providing an enriched patient population with a high enough background incidence to practically allow measuring the ability of an iPBP4 adjuvant to reduce osteomyelitis (Zhang et al., 2023). Corresponding study estimates to measure the efficacy of an iPBP4 adjuvant (added to the standard-of-care antibiotics) can be easily modeled based on improving a 16% infection rate, depending on the expected reduction in osteomyelitis, to include 248 patients per arm (expected 50% reduction by iPBP4) to 1, 131 patients per arm (expected 25% reduction by iPBP4), based on 80% power, two-sided test α = 0.05. Consequently, a path forward for the clinical development of iPBP4 adjuvants could lie in reducing severe open fracture-associated osteomyelitis.

In terms of the clinical development of *S. aureus* PBP4 inhibitors as antibiotic resistance breakers, one may anticipate that the clinical path may be more straightforward and could be modeled on the successes of β-lactam/β-lactamase inhibitor combinations. Such combinations include amoxicillin-clavulanate, piperacillin-tazobactam, ceftazidime-avibactam, and meropenem-vaborbactam, and follow the same premise: the second molecule maintains the β-lactam activity of the first by inhibiting resistance determinant(s) (Carcione et al., 2021). In that regard, there is growing appreciation that PBP4 overexpression leads to high-level resistance not only to the fifth generation cephalosporins but also to a broader range of β-lactams and gives rise to a noncanonical PBP2A-independent MRSA phenotype. As a result, it is becoming increasingly clear that *mecA* (PBP2A) based detection diagnostics, which do not take into account PBP4, are likely to underestimate MRSA rates and/or dissemination, necessitating diagnostic revision and a firmer understanding of PBP4-mediated β-lactam-resistant patient populations that could best be targeted in a clinical development program. As alluded to above, there is a clear precedent for revising MRSA diagnostics, as Cheung and colleagues have established that PBP4 is a major driver of community-acquired MRSA (Memmi et al., 2008).

In the absence of a more refined understanding of the bona fide frequency of PBP4-mediated MRSA across infection settings to guide a clinical development plan, a simple extension of the β-lactam and resistance-breaker (i.e., iPBP4) strategy could be considered. In this scenario, one would expect the β-lactam to retain antibacterial activity despite high levels of PBP4 due to co-administration of an iPBP4. However, the question is which β-lactam antibiotic would be best paired with the iPBP4? Ideally, the antibiotic would be a contemporary β-lactam with a modest frequency of resistance in the patient population, thereby providing a reasonable opportunity to measure the restoration of the antibiotic’s antimicrobial efficacy in the presence of iPBP4 and improve patient outcomes. To that end, the latest generation cephalosporins, ceftobiprole and ceftaroline, are approved and used to treat acute bacterial skin and skin structure infections (SSSI), and resistance to both antibiotics is mediated by PBP4 overexpression. Thus, it may be tempting to consider a clinical development program designed to assess the ability of iPBPs to improve the efficacy of either antibiotic in the SSSI setting by overcoming the corresponding resistance. However, antibiotic susceptibility surveys of SSSI clinical isolates indicate ceftaroline and ceftobiprole resistance is only 1.5% and 0.6%, respectively, with similar resistance rates for both antibiotics for other infection times (Sader et al., 2023; Flamm et al., 2020). Thus, as in the case of reducing TJR-associated osteomyelitis, a clinical program designed to improve the therapeutic performance of either agent is likely unreasonable, due to the sheer numbers of patients that would be required to confidently measure an improvement in treatment by either ceftaroline or ceftobiprole, due to co-administration of an iPBP4 aimed at reversing corresponding resistance.

From these perspectives, currently, the most straight forward and reasonable clinical development path for a PBP4 inhibitor is likely to be in the context of its use as an adjuvant that is co-administered with standard of care antibiotics for reducing Type III open tibial fracture associated osteomyelitis. However, it is fully recognized that this is likely to change. As antibiotic resistance to ceftaroline and/or ceftobiprole mounts, so too will the opportunity to develop clinical pathways to test the safety and efficacy of iPBP4s in breaking corresponding resistance. Given the early stage of development of the current phenyl urea and benzothiophene chemical classes of iPBP4s, combined with the importance of the fifth generation cephalosporins to US health and the lack of new classes of antibiotics in the drug discovery pipeline, it seems worthwhile to continue their development as a preemptive means to preserve the efficacy of these two very valuable antibiotics.

## 8 Conclusions

*S. aureus* PBP4 has only recently been recognized to modulate the organism’s ability to cause disease. Hence, the protein has historically received much less attention and is poorly characterized in comparison to *S. aureus* essential penicillin-binding proteins and/or PBP2A. Emerging studies are beginning to shed light on the regulatory networks that modulate PBP4 expression, and it seems likely that such studies, combined with the high degree of protein conservation across *S. aureus* lineages, will further illuminate the protein’s importance in pathogenic processes (Satishkumar and Chatterjee, 2026). Moreover, it also seems likely that such work will have expanded significance, given that the role of PBP4 in PBP2A-independent high-level β-lactam resistance is not limited to *S. aureus. Enterococcus faecalis* PBP4 overexpression and/or mutations increase ceftobiprole resistance (Lazzaro et al., 2021). In *Pseudomonas aeruginosa*, mutations of non-essential low molecular mass PBP4 trigger a high level of β-lactam resistance by AmpR-dependent overexpression of the AmpC β-lactamases (Moya et al., 2009).

While it is not clear whether PBP4-mediated β-lactam resistance is directly linked to the protein’s transpeptidase activities, mutations within the *S. aureus* PBP4 transpeptidase active site serine residue eliminate resistance, suggesting these functions are directly linked. Similarly, the ability of PBP4 to promote the formation of highly cross-linked PG presumably provides the architectural stability and integrity needed to maintain cellular viability during bacterial deformation into nanopore-sized OLCNs, and strains lacking the active-site serine residue fail to colonize OLCNs *in vitro*. Thus, while the molecular mechanisms by which PBP4 mediates *S. aureus* antibiotic resistance and OLCN invasion remain to be determined, it is intriguing to consider that they may be directly linked; hence, it may be possible to develop dual-functional PBP4 inhibitors that could be used as antibiotic adjuvants to reduce (or reverse) β-lactam resistance and recurring osteomyelitis.
